# Layered Gradient Grain Structure Enhances Mechanical Properties of Ultra-Thin Copper Foil

**DOI:** 10.3390/ma19030520

**Published:** 2026-01-28

**Authors:** Xixi Wang, Jing Wei, Jian Huang, Chun Yang, Yixin Luo, Yanle Huang, Ning Song, Yuhui Tan, Hongguang Yang, Sujie Qi, Xiaowei Fan, Yunzhi Tang

**Affiliations:** 1 Gannan Laboratory, 1958 Hakka Avenue, Ganzhou 341000, China; 2 Jiangxi Province Key Laboratory of Functional Crystalline Materials Chemistry, School of Chemistry and Chemical Engineering, Jiangxi University of Science and Technology, Ganzhou 341000, China; 3 State Key Laboratory of Nonferrous Structural Materials, Jiangxi University of Science and Technology, Ganzhou 341000, China; 4 Jiujiang Defu Technology Co., Ltd., No. 15, Shunyi Road, Automobile Industrial Park, Jiujiang 332000, China

**Keywords:** electrolytic copper foil, additives, gradient grains, grain refinement strengthening, mechanical properties

## Abstract

Traditional homogeneous copper foils suffer from a trade-off between strength and ductility, while gradient or heterogeneous structures are mostly based on deformation processing, making it difficult to achieve controllable construction within a thickness of ≤10 μm. This study aims to directly construct a layered structure with a “fine–coarse–fine” (A-B-A) gradient grain distribution, denoted as 3L-ABA in an 8 μm copper foil via direct current electrodeposition, which utilizes composite additives to regulate electrochemical polarization and nucleation modes. Through systematic characterization and mechanical testing, it was found that the 3L-ABA copper foil exhibits a tensile strength of 604 ± 18 MPa, an elongation of 3.6 ± 0.25%, and low surface roughness Rz of 0.46 μm. Microscopic mechanism analysis demonstrates that the gradient structure achieves synergistic strengthening and toughening through surface fine-grain strengthening, intermediate coarse-grain coordinated plastic deformation, combined with dislocation density and twin strengthening. Electrochemical tests confirm that Additive A (containing collagen, bis-(3-sulfopropyl)-disulfide (SPS), thiourea and 2-mercapto-5-benzimidazolesulfonic acid sodium salt (2M5S)) induces strong cathodic polarization, promoting instantaneous nucleation and grain refinement, whereas Additive B (containing collagen and bis-(3-sulfopropyl)-disulfide (SPS) shows weaker polarization and promotes grain growth. This research provides a scalable electrodeposition solution for the microstructural design and performance regulation of ultra-thin copper foils.

## 1. Introduction

Lithium-ion battery copper foil, as the conductor material for the negative electrode current collector, is widely used in new energy vehicles and energy storage fields [1,2]. In recent years, with rapid technological advancement, copper foil has been developing towards ultra-thinning [3] and structural complexity [4,5]. However, when the foil thickness is reduced below 10 μm, its mechanical properties deteriorate sharply, making it prone to fracture during electrode winding and charge–discharge volume expansion, potentially leading to safety hazards such as short circuits [6,7,8]. Electrodeposition, as the mainstream preparation process for copper foil, can regulate grain nucleation and growth through additives and process parameters, thereby influencing its microstructure and properties [9,10]. In metal structural property theory, according to the Hall–Petch relationship, grain refinement strengthening can significantly enhance strength but often sacrifices ductility; coarse grains, while beneficial for ductility, lead to a reduction in strength [11]. This is attributed to the poor dislocation accommodation capacity in small grains, causing a substantial decrease in ductility. Conversely, increasing the crystalline grain size only contributes to coarse-grain plasticity while weakening strength, representing the so-called strength–ductility trade-off [12,13].

Recent research efforts have attempted to synergistically improve the strength and toughness of metallic materials by constructing gradient or heterogeneous structures. For instance, Dun et al. [5] achieved a high elongation of 22.68% using polymer layer selection and surface metal deposition combined with interface modification, but the tensile strength remained low at 297 MPa. Huang et al. [14] obtained copper foil with low surface roughness (Rz = 1.48 μm) by adding hydroxyethyl cellulose (HEC). Although the tensile strength was ≥528 MPa, the elongation dropped to ≤2.5%. Liu et al. [15] achieved a relatively high elongation of 6.5% in copper foil using gelatin additives combined with carrier-assisted electrodeposition, but the tensile strength was only ~340 MPa. Current research on thin-layer materials has focused on laminated layers, achieving excellent high-strength and high-ductility properties in copper foils through gradient and heterogeneous structures. Shin et al. [16] obtained multilayer laminated electrolytic copper foil by adjusting additive concentration and modulating current density, achieving grain size control from 1.0 to 10 μm. The tensile strength of fine grains was relatively high but did not exceed 360 MPa, while the elongation of coarse grains increased with foil thickness: ≤6% for 20 μm thickness and ≤30% for 100 μm thickness. Wang [17] and Zhou [18] et al. constructed heterogeneous structures in thick plates (>400 μm) via Equal Channel Angular Pressing (ECAP, a typical severe plastic deformation technology) and Dynamic Recrystallization Annealing (DRA) techniques, respectively, achieving a good balance of strength and ductility. Heterogeneous or gradient structures can enhance material strength without compromising ductility [19,20]. However, most existing studies focus on obtaining homogeneous fine or coarse-grained structures using a single additive system. Research on the direct electrodeposition of layered gradient grain structures with well-defined layers within an ultra-thin scale (8 μm) through composite additive systems and programmed current density control remains scarce [4].

Based on this, we successfully prepared an 8 μm ultra-thin copper foil with a multilayer gradient grain structure via direct current electrodeposition, achieving simultaneous improvement in both strength and toughness. The study further discusses the intrinsic relationship between the microstructure and mechanical properties of gradient grain copper foils, as well as the key mechanism of additives in electrodeposition, through microstructural observation and electrochemical test analysis.

## 2. Materials and Methods

**Figure 1:**
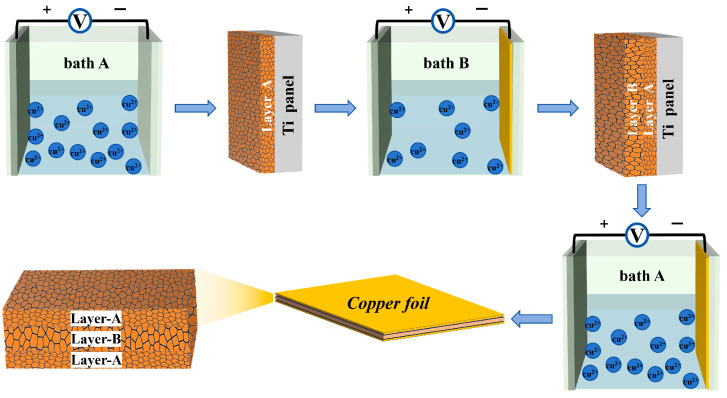
Process flow diagram for multilayer copper foil preparation.

The preparation process flow for the layered gradient grain structure copper foil is shown in Figure 1. A custom planar electrodeposition setup was used, with a pure titanium plate as the cathode and an iridium-coated titanium plate as the anode. The distance between the electrodes was maintained at 10 cm. Copper foil with a thickness of approximately 8 μm was deposited under direct current (DC). At room temperature, the copper ions and the additive solution are dispersed uniformly by introducing the bubble flow to stir the bath.

The plating bath compositions are shown in Table 1. All reagents used in the experiment were of analytical grade, and the water for plating solution preparation was distilled water. The three-layer 3L-ABA copper foil with grain variation was fabricated through a three-step process: first, copper foil layer A was deposited in plating solution A at 30 A/dm^2^ constant current density; then, layer B was deposited in solution B at 3 A/dm^2^ constant current density; and finally, layer A was deposited again in solution A at 30 A/dm^2^ constant current density. Additionally, single-layer 1L-A and 1L-B copper foils were separately prepared, and their microstructural properties were compared.

For the experiment, we prepared 8 μm thick 3L-ABA, 1L-A single-layer copper foil and 1L-B single-layer copper foil by electrodeposition. The current densities and deposition times for each step were selected based on preliminary experiments to precisely control the thickness of individual layers (2 μm for A-layers, 4 μm for the B-layer) and to ensure the formation of the intended fine- or coarse-grain structures. Then, the copper foil was peeled off from the cathode plate, rinsed with deionized water, and air-dried.

Tensile strength and elongation were measured at room temperature using an EM-6.203-L (Sakai, Japan) universal mechanical testing machine, with at least three valid specimens tested per sample. The specimen dimensions were 150 mm × 12.7 mm, with a gauge length of 50 mm and a constant strain rate of 2 mm/min. Testing followed the GB/T 29847-2013 standard [21]. Surface three-dimensional (3D) profiles were analyzed, and surface roughness was obtained using a laser confocal microscope (OLYMPUS-YTL336, Olympus Corporation, Tokyo, Japan). Surface morphology was characterized using a scanning electron microscope (SEM, MLA650F, FEI, TESCAN, Brno, Czech Republic) operating at 10 kV. Crystal structure was characterized using an X-ray diffractometer (XRD, Bruker D8 Advance, Dandong Tongda Technology Co., Ltd., Dandong, China). Grain size and crystal orientation distribution were measured using an electron backscatter diffractometer (Nordlymax3, Oxford Instruments plc, Oxford, UK) with a step size of 0.1 μm. Grain size analysis was performed using AZtec Crystal 2.1 software. Electrochemical measurements were conducted using an electrochemical workstation (660E, CHI, Shanghai Chenhua Instrument Co., Ltd., Shanghai, China) in a three-electrode system. A pure titanium rod (diameter 1 mm) served as the working electrode, a high-purity platinum sheet (15 mm × 15 mm) as the counter electrode, and a saturated calomel electrode (SCE) as the reference electrode. Prior to each measurement, the working electrode surface was polished with 0.3 μm particle size Al_2_O_3_ polishing powder and then cleaned with deionized water. In linear sweep voltammetry (LSV) tests (used to analyze deposition potential and kinetics), the starting voltage was set to −0.1 V, the ending voltage to −0.5 V, and the scan rate to 10 mV/s. In galvanostatic (GM) tests (used to monitor potential change at constant current), the current density was set to 30 A/dm^2^ for bath A and 3 A/dm^2^ for bath B. Additives including collagen, bis-(3-sulfopropyl)-disulfide (SPS), thiourea (TU), and 2-mercapto-5-benzimidazolesulfonic acid sodium salt (2M5S) were sequentially added every 200 s. Changes in electrode potential were continuously recorded. All experiments were conducted at room temperature.

## 3. Results and Discussion

### 3.1. Surface Property Analysis

**Figure 2:**
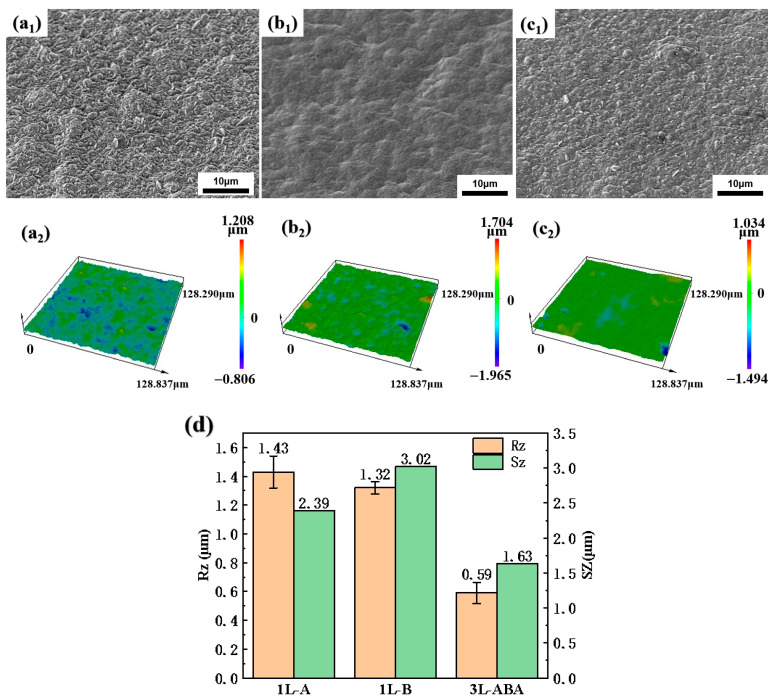
Copper foil surface morphology: (**a_1_**–**c_1_**) SEM images of 1L-A, 1L-B, 3L-ABA; (**a_2_**–**c_2_**) are CLSM images of 1L-A, 1L-B, 3L-ABA; (**d**) is the histogram of copper foil surface roughness distribution.

Surface roughness refers to the unevenness of copper foil surface with small spacing and tiny peaks and valleys. The degree of such surface roughness is usually quantified by the parameters Rz and Sz. Rz represents the distance between the peak and the valley bottom of a profile. Sz represents the vertical distance between the highest peak and the lowest valley on a three-dimensional surface. Typically, reducing roughness is key to improving copper foil surface morphology [22], with additives significantly influencing the crystal structure [23,24]. A composite additive can effectively inhibit grain growth and achieve low roughness [25]. To reduce surface roughness and ensure the effective thickness of each copper foil layer, we employed different additives: A (containing collagen, SPS, thiourea, and 2M5S) and B (containing only collagen and SPS). The surface morphology changes of 1L and multilayer 3L structured copper foils observed by SEM are shown in Figure 2. The 1L-A surface exhibits distinct spindle-shaped particles that are fine with relatively small spacing, and the difference between surface profile peaks and valleys is small (Figure 2(a_1_)). Based on Confocal Laser Scanning Microscopy (CLSM) observations, the roughness Rz at this point is 1.01 μm. The 1L-B surface clearly shows large particles with significant undulations (Figure 2(b_1_)), and the roughness Rz is 1.38 μm. This indicates that the Additive A composition is more effective in reducing surface roughness compared to Additive B. The 3L-ABA surface appears flatter with no obvious undulations (Figure 2(c_1_)), and the roughness Rz is 0.46 μm, lower than that of 1L-A. This is primarily due to the influence of bath component depletion with increasing deposition thickness. The outer A-layers in 3L are only 2 μm thick, whereas the 1L-A foil is 8 μm thick. As the electrodeposition thickness increases, the concentration of additive components in the bath gradually depletes, affecting grain size and its resulting roughness difference. Consequently, the A-layer of the prepared 3L-ABA consumes only one fourth of the bath components compared to the 1L-A copper foil, resulting in lower surface roughness compared to 1L-A.

Meanwhile, as shown in Figure 2d, the surface roughness results across the three copper foils showed consistency with SEM measurements. 3L-ABA exhibited the lowest surface roughness Sz at 0.92 μm (Figure 2(c_2_)). The reduction of roughness Rz mainly stems from controlling the thickness uniformity of each layer and ultimately accurately obtaining the grain structure of each layer with a thickness of 2 μm. Especially when the single-layer grain structure of 1L-A and 1L-B is superimposed on three layers of 3L-ABA, it is even more necessary to maintain the effective thickness of each layer.

### 3.2. Mechanical Performance Analysis

**Figure 3:**
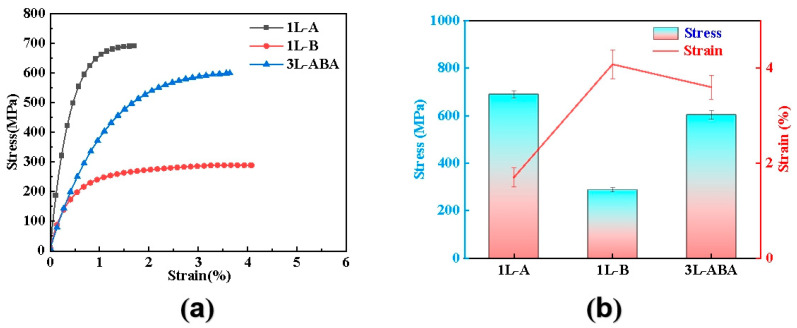
(**a**) Copper foil tensile engineering stress–strain curves; (**b**) stress–strain histogram of copper foil tensile engineering.

The tensile strength and elongation of copper foil were tested at room temperature under the GB/T 29847-2013 standard (Figure 3). For homogeneous copper foils, the figure shows a clear trade-off between tensile strength and ductility. In single-layer foil tests, the tensile strength of 1L-A is 690 ± 15 MPa, but its elongation is only 1.7 ± 0.2%; the tensile strength of 1L-B is as low as 289 ± 10 MPa, with an elongation of 4.08 ± 0.3%. For the gradient grain-structured 3L-ABA, the tensile strength reaches 604 ± 18 MPa while maintaining an elongation of 3.6 ± 0.25%. In Figure 3a, the 3L-ABA structure exhibits a more gradual strain hardening stage during tensile testing, suggesting that its internal gradient interfaces effectively coordinate plastic deformation and delay necking, thereby maintaining good deformation capability while enhancing strength. These results confirm the effectiveness of the layered gradient structure in breaking the strength–ductility trade-off in ultra-thin copper foils.

### 3.3. Crystal Structure and Texture Analysis

**Figure 4:**
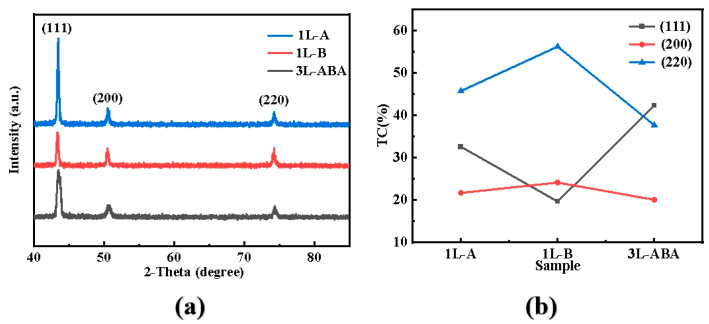
(**a**) XRD spectra of 1L-A, 1L-B, and 3L-ABA copper foil surfaces; (**b**) texture coefficients.

The crystal structures of the various layered copper foils were analyzed using XRD, with results shown in Figure 4a. All foils exhibit three distinct diffraction peaks corresponding to the (111), (200), and (220) planes. The preferred orientation of the diffraction peaks was quantified using the texture coefficient (T*_C_*). T*_C_* quantitatively characterizes the orientation distribution of specific crystal planes in copper foil by measuring the relative ratios of XRD diffraction intensities calculated from the XRD peak intensities and standard values as shown in the following formula:

(1) $$T_{C}=\frac{{Ι}_{\left(hkl\right)}∕{Ι}_{0(hkl)}}{\sum_{i=1}^{n}{Ι}_{\left(hkl\right)}∕{Ι}_{0(hkl)}}$$

Here, Ι_(hkl)_ and Ι_0(hkl)_ represent the diffraction peak intensities of the copper foil sample and the standard copper sample, respectively. Ι_0(hkl)_ uses the intensity values from the standard powder diffraction card (JCPDS No. 04-0836). The n is the number of diffraction peaks in the XRD spectrum. A higher texture coefficient indicates stronger preferred orientation growth of the copper foil. According to Formula (1), the calculated texture coefficients for the crystal planes are shown in Figure 4b. Except for 1L-B, the other layered foils show relatively high orientation for both (111) and (220) planes. High (111) texture can significantly enhance the strength of copper foil through synergistic interaction with “nanotwins” [26]. The orientation of copper foil was changed from (111) to (220) by using the composite additive and electrodeposition method, which greatly improved the elongation [27]. And the surface diffusion rate of (220) high-orientation copper is superior to that of other crystal planes [28]. The single-layer 1L-A has a T_C_ (111) of 32%, and 1L-B has a T_C_ (220) of 56%, which explains well the better tensile strength of 1L-A and the better elongation of 1L-B, consistent with the tensile test results in Figure 3. However, compared to the single-layer structures, the three-layer structure more significantly regulates the grain organization. In 3L-ABA, the (220) plane preferred orientation is substantially reduced, while the (111) plane orientation is significantly enhanced, with a T_C_ (111) of 42%. The contribution of texture to strength can be attributed to the release of residual internal stress in the electrolytic copper foil, generating a large number of dislocations and metastable structures within the grains that hinder deformation during tensile testing [29,30], ultimately affecting the mechanical properties of the copper foil.

The twin density (β) is quantitatively determined by X-ray diffraction analysis. The twin density (β) is defined as the proportion of twin boundaries on the (111) crystal plane, and the calculation formula is as follows:

(2) $$\beta =\frac{\left[∆C.G.({2\theta }_{111})-∆C.G.({2\theta }_{200})\right]}{(11\tan\theta_{111}+14.6\tan\theta_{200})}$$

Among them, ∆C.G.(2θ_111_) and ∆C.G.(2θ_200_) are, respectively, the angular deviations of the centre of gravity of the (111) and (200) X-ray diffraction peaks relative to their maximum values. Using Formula (2), the calculated twin densities for 1L-A, 1L-B, and 3L-ABA are 0.236%, 0.009%, and 0.101%, respectively. Thus, 1L-A has the highest twin density, 1L-B has the lowest, and the 3L-ABA sample’s twin density is lower. This is because the middle layer of 1L-B consists of coarse grains with low twin density, which consequently affects the overall 3L-ABA, making it lower than 1L-A. Twins enhance material strength through the “twin boundary strengthening effect” by hindering dislocation movement [31]. The intermediate twin density of 3L-ABA indicates that its strengthening mechanism involves the synergistic effect of twins, dislocations, and the gradient structure.

### 3.4. Microstructure Analysis

#### 3.4.1. Grain Size Gradient Analysis

**Figure 5:**
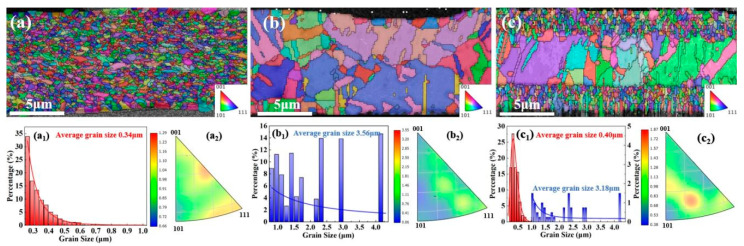
EBSD electron backscatter diffraction patterns of copper foil cross-sections: (**a**) 1L-A; (**b**) 1L-B; (**c**) 3L-ABA; copper foil grain size statistics: (**a_1_**) 1L-A; (**b_1_**) 1L-B; (**c_1_**) 3L-ABA; inverse pole figures: (**a_2_**) 1L-A; (**b_2_**) 1L-B; (**c_2_**) 3L-ABA.

Among the influences on the mechanical properties of metallic materials, fine-grained strengthening and coarse-grained shaping play a major role. For this purpose, EBSD was used for microstructure analysis in the designed layered gradient grain structure, and the results are shown in Figure 5. High current density (30 A dm^−2^) leads to an increase in deposition overpotential. The higher overpotential during the electrodeposition process results in a small-grain structure with fine twinned spacing [19,32], promoting the formation of a fine-grain microstructure in the 1L-A monolayer, with an average grain size of 0.34 μm. The overpotential generated during the deposition process at a low current density (3 A/dm^2^) is lower, the cathodic polarization is weakened, and the corresponding grain growth during recrystallization occurs, forming a relatively large-sized grain structure [33], which in turn affects the formation of a coarse-grain microstructure in the 1L-B monolayer, with an average grain size of 3.56 μm. The average grain sizes of 1L-A and 1L-B differ by more than ten times, forming a significant difference [16]. Due to the influence of the 1L-A fine-grain strengthening mechanism, the corresponding square point curve results in Figure 3, exhibit mechanical properties of high strength and grain size weak ductility. However, due to the influence of the 1L-B coarse-grained plasticity mechanism, the corresponding spherical point curve results in Figure 3 exhibit mechanical properties of low strength and high ductility. High-performance metallic materials are designed through interface regulation, where dual interfaces further disperse stress, enabling a synergistic ‘strengthening-plasticity’ mechanism [34] and the optimization of final mechanical properties. Thus, the three-layer structure 3L-ABA was designed by combining the fine-grained layer of 1L-A with the coarse-grained layer of 1L-B, resulting in a sandwich-like layered grain structure of “fine-grained–coarse-grained–fine-grained”. The cross-section of the copper foil was analyzed by EBSD. It still showed that the surface grain size was close to that of 1L-A, which was 0.40 μm. The middle layer is 3.18 μm. This gradient design allows surface fine grains to inhibit crack initiation (fine-grain boundaries hinder crack propagation), thus improving fatigue resistance [13]. The coarse grains in the middle layer can release plastic strain through grain sliding, avoiding brittle fracture and reducing stress concentration [29]. It can be seen from the reverse polarity diagram that the 1L-A foil (Figure 5(a_2_)) is more inclined towards the (111) orientation. Due to the close-packed crystal planes of copper, (111) has a stronger atomic binding force between the close-packed crystal planes, making it more difficult for the crystal planes to slip. A higher external force is required to trigger plastic deformation or fracture. In addition, the 1L-A foil exhibits a higher preferred orientation in the transition region between (001) and (111). The cross-section of the continuous columnar polycrystalline copper foil has the original preferred orientation of <001>, and the deformation energy storage of the <001> orientation texture is lower than that of the <111> orientation. This means that the work hardening rate of the (001) orientation is low, and its effect on enhancing tensile strength is relatively weak, which is beneficial to elongation [35]. The grains with different orientations deform collaboratively through their respective slip systems when subjected to stress, preventing the concentration of deformation in a single orientation. This allows the material to undergo greater plastic deformation before fracture. Moreover, the random orientation reduces the issue of deformation incoordination caused by excessive or insufficient orientation differences between grains, further optimizing the plastic deformation [36]. The random orientation in the 1L-B foil (Figure 5(b_2_)) ensures that when the copper foil deforms under force, more grains can participate in the coordinated deformation, avoiding local stress concentration, which is conducive to improving the elongation. The high selective orientation of the 3L-ABA foil (Figure 5(c_2_)) is located in the transition zone between (110) and (111) centers in the reverse polarity diagram. The high (111) orientation endows the copper foil with a higher tensile strength, while the high (110) orientation results in a better elongation rate. Moreover, the overall color distribution in the diagram is relatively uniform. This uniform distribution of transitional orientation enables the stress to be evenly transmitted within the copper foil without obvious mechanical weak areas, which can delay the occurrence of fracture (supporting high tensile strength) and provide room for continuous deformation [37], ultimately demonstrating an excellent balance of “strength and plasticity” on a macroscopic scale.

#### 3.4.2. KAM Analysis

KAM refers to the calculation of the average orientation difference between a given measured pixel and its adjacent pixels. GND refers to the dislocations that are indispensable for accommodating the inhomogeneity of plastic deformation inside the material and maintaining the lattice orientation gradient as well as deformation compatibility. The kernel average misorientation (KAM) value reflects local lattice distortion and is approximately proportional to the geometrically necessary dislocation (GND) density [38,39]. Therefore, under low KAM conditions (~0.4°), statistical analysis of KAM can also reflect dislocations in the copper foil. Additionally, the GND density can be calculated using the following formula:

(3) $$\rho_{GND}=\frac{{2KAM}_{ave}}{\mu B}$$

**Figure 6:**
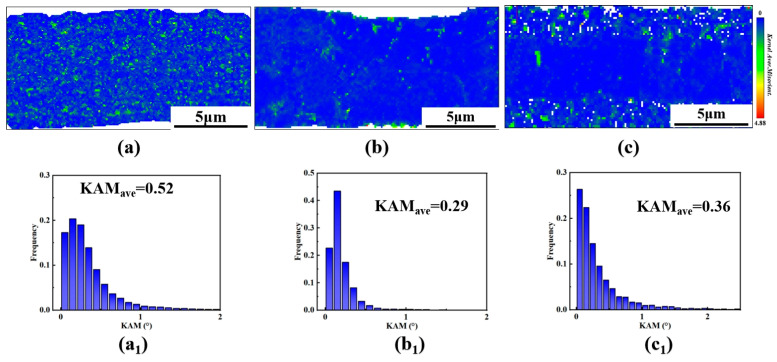
(**a**–**c**) KAM distribution maps; (**a_1_**–**c_1_**) KAM histograms.

Here, *μ* represents the EBSD scanning step size (*μ* = 1.0 × 10^−7^ m), KAM*_ave_* is the average KAM value corresponding to the sample, and B is the Burgers vector (for pure copper, B ≈ 0.256 × 10^−9^ m). The calculated KAM*_ave_* values for 1L-A, 1L-B, and 3L-ABA are 0.52°, 0.29°, and 0.36°, respectively. The corresponding *ρ*_GND_ are 2.77 × 10^16^/m^2^, 1.53 × 10^16^/m^2^, 1.9 × 10^16^/m^2^, respectively. Combined with the KAM statistical chart analysis in Figure 6, the results show that 3L-ABA has a high proportion of grains with KAM values below 0.4°, indicating less local stress concentration. Compared to 1L-A, its dislocation density is lower but still significantly higher than that of 1L-B, and dislocations are mainly distributed at the interlayer interfaces. The fact that 3L-ABA maintains higher strength than 1L-A is attributed to the back stress strengthening induced by heterogeneous interlayer interfaces [40]. During tensile deformation, the strain incompatibility between the soft (coarse-grained) and hard (fine-grained) regions leads to the accumulation of high-density geometrically necessary dislocations (GNDs) at the interfaces. The resulting significant back stress directly strengthens the coarse-grained region, contributing to strength that surpasses that of the coarse-grain homogeneous structure [41]. Additionally, its strengthening mechanism may also be attributed to the dislocation starvation mechanism [19]. The high stacking fault energy inhibits the decomposition of full dislocations into incomplete dislocations, facilitating dislocation escape from the surface. This enhances the dislocation starvation mechanism, resulting in a dislocation-scarce state for small-sized copper nanostacks during plastic deformation. The deformation is predominantly elastic until new dislocations nucleate [42]. Using high current density to generate the fine-grain A-layer structure corresponds to requiring high stress/strain energy to activate new dislocations, thus exhibiting high strength even at a low dislocation density. KAM values are closely related to crystal deformation. KAM value reflects local orientation differences. Higher values indicate more significant stress concentration within the crystal and higher GND density (ρ_GND_) [43]. Lower KAM values indicate more uniform deformation within grains, further enhancing structural stability [44], as show in Figure 6. This mechanism is more pronounced in materials without columnar twins, consistent with the twin distribution characteristics of the layered gradient grain structure copper foil [45].

**Figure 7:**
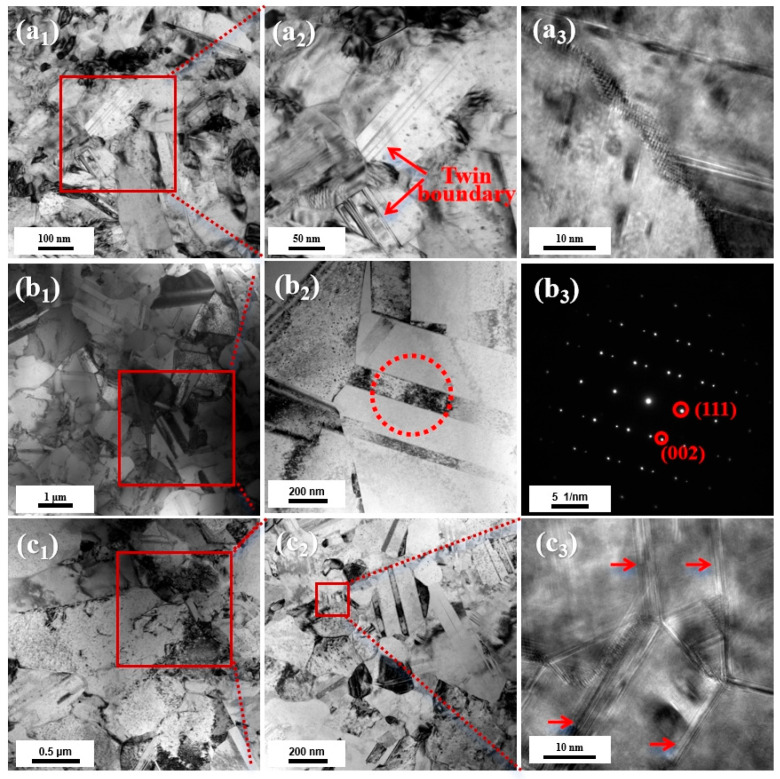
Transmission electron microscopy (TEM) images of each copper foil: (**a_1_**) 1L-A low-power image; (**a_2_**) a_1_ red-boxed magnification; (**a_3_**) 1L-A high-resolution TEM (HRTEM) image; (**b_1_**) 1L-B low-power image; (**b_2_**) b_1_ red-boxed magnification; (**b_3_**) b_2_ red-circle selected-area diffraction (SADP) image; (**c_1_**) 3L-ABA low-power image; (**c_2_**) c_1_ red-boxed magnification; (**c_3_**) 3L-ABA high-resolution TEM (HRTEM) image (The red arrow indicates the dislocation line).

TEM was used to analyze the microstructural organization of the copper foils. Typically, higher current density increases overpotential, thereby increasing electrochemical polarization, leading to the formation of smaller grains and twin spacing in the copper foil [32]. Figure 7 shows TEM images of the 1L-A, 1L-B, and 3L-ABA copper foils. It can be seen that the 1L-A copper foil contains a large number of fine grains (in the size range of 100–300 nm). Within some of these fine grains, a high density of twins can be observed (Figure 7(a_1_)). These twins are small, with some twin boundary spacings as small as 10 nm (Figure 7(a_2_)). The grain size in 1L-B copper foil is coarse (Figure 7(b_1_)), and most of the grain sizes are greater than 1 μm. Twins can also be observed within the coarse grains (Figure 7(b_3_)). Compared with 1L-A copper foil, the twins within the grains of 1L-B copper foil are coarser and fewer in number. There are two microstructure regions with significant differences in grain size in 3L-ABA copper foil, namely, the coarse-grained microstructure region and the fine-grained microstructure region. The coarse-grained microstructure region is similar to that of 1L-B copper foil, with only a small number of twins existing within the coarse grains (Figure 7(c_2_)). In the fine-grained structure region, some fine grains have high-density nanoscale twins (Figure 7(c_3_)). The twinning density in the fine-grained region is much higher than that in the coarse-grained structure region.

### 3.5. Electrochemical Characteristic Analysis

#### 3.5.1. Galvanostatic (GM) Analysis of Additive Synergistic Effects

**Figure 8:**
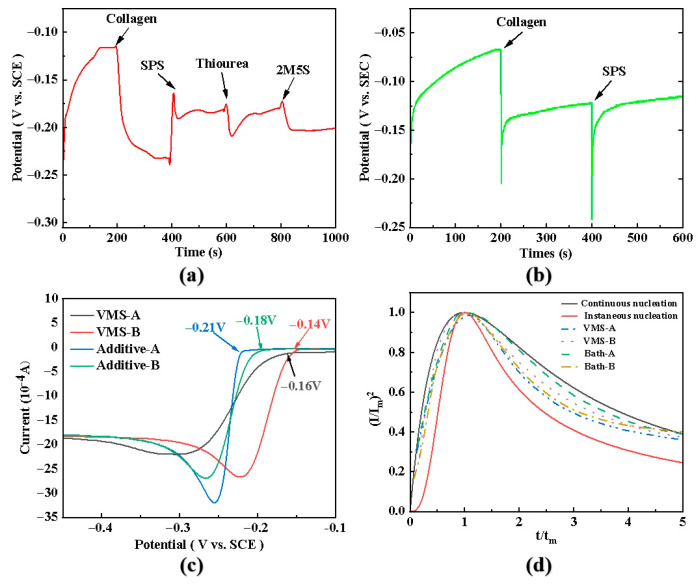
(**a**,**b**) GM curves of composite Additives A and B, respectively; (**c**) LSV curves; (**d**) the relationship curve between the recovery current density (j_p_) and the square root of the scanning rate (ν^1/2^).

To investigate the instantaneous effects of each additive, galvanostatic chronopotentiometry tests were conducted, as shown in Figure 8a. Initially, in the base bath VMS-A (VMS, Voltammetry Measurement Solution, containing only Cu^2+^, H_2_SO_4_, and HCl, without additives), the starting potential was approximately −0.25 V, stabilizing later at −0.11 V. This is because the cathode deposition interface possesses a Helmholtz layer, requiring a larger activation energy to penetrate this interfacial film layer [46], thereby providing Cu^2+^ from the solution bulk for reduction and deposition, reaching a double-layer equilibrium state. After stabilization, the potential remains at −0.11 V. Upon adding collagen, the potential significantly shifts negatively to −0.23 V, exhibiting a strong polarization effect, indicating effective inhibition of copper deposition. Subsequently, adding bis-(3-sulfopropyl)-disulfide (SPS) shifts the potential positively to −0.16 V, showing a depolarization effect and creating an antagonistic interaction with collagen, demonstrating a significant depolarizing effect. After adding thiourea (TU), the potential change is complex: a brief positive shift followed by a negative shift to −0.21 V, ultimately stabilizing at −0.18 V. This is related to its molecular structure containing both polarizing (amino) and depolarizing (mercapto) groups [24]. Finally, adding 2-mercapto-5-benzimidazolesulfonic acid sodium salt (2M5S) shifts the potential negatively again to −0.20 V and remains stable, indicating its sustained polarizing effect. The four additives in bath A collectively exhibit a net polarization effect, maintaining a stable deposition potential of approximately −0.20 V, favoring the formation of dense fine grains.

In contrast, for bath B, the GM curve in Figure 8b shows that after adding only collagen and SPS, the deposition potential stabilizes at −0.12 V. Its degree of polarization is much lower than that of bath A (more positive potential). This is related to factors such as the higher Cu^2+^ concentration in bath B and the relatively higher content of collagen, ultimately leading to grain coarsening. Compared with bath VMS-A, bath VMS-B has a higher Cu^2+^ concentration, and the collagen content is relatively higher than that of SPS. The molecule contains a large number of amino groups, increasing coordination with Cu^2+^, overall exhibiting a polarization effect, increasing overpotential, and masking the polarizing effect of the mercapto groups in the SPS molecule. Ultimately, the additive-containing bath B maintains a stable potential of −0.12 V, which is far lower (more positive) than the overpotential of additive-containing bath A. Therefore, the obtained 1L-B copper foil has coarse grains. Meanwhile, the stable deposition potential without fluctuation results in a dense and smooth surface for the 2 μm thick copper foil grain structure.

#### 3.5.2. Linear Sweep Voltammetry (LSV) Analysis of Bath Polarization Effects

The potential variation in each electroplating solution during the electrodeposition process was analyzed by electrochemical tests, with results shown in Figure 8c. Compared to their respective base baths (VMS), after adding composite additives, both bath A and bath B show a negative shift in their starting deposition potentials, with overpotentials increasing by 0.05 V and 0.04 V, respectively. This confirms that the additives can inhibit deposition, with Additive A having stronger polarization capability. Notably, the cathodic peak current for bath A is much higher than that of other conditions. This indicates that under strong polarization, its interfacial reaction kinetics change significantly, more favoring the formation of a large number of nuclei. Furthermore, the peak current can reflect the reaction rate under given conditions. Larger overpotential leads to smaller critical nucleation size during electrodeposition, making the nucleation process more favorable compared to the growth process [27,47].

#### 3.5.3. Nucleation Mode and Deposition Kinetics

The dimensionless equation (I/I_m_)^2^ − (t/t_m_)^2^ was used to fit the potentiostatic transient curves for baths VMS-A, bath B, bath A, and VMS-B, as shown in Figure 8d. Based on theoretical instantaneous and continuous nucleation curves, under different deposition potentials, bath A exhibits a nucleation mode more closely resembling instantaneous nucleation, which favors high-density nucleation and grain refinement. Conversely, bath B exhibits a nucleation mode more closely resembling continuous nucleation, which favors grain coarsening [47,48].

By regulating current density combined with additives, nucleation can be accelerated, and grain growth inhibited, effectively refining grains [32]. Additive Group A, through polarization combined with high current density, promotes bath A to exhibit an instantaneous nucleation mode, which increases the number of nuclei, inhibits grain growth, and thus achieves grain refinement. This fine-grain strengthening gives 1L-A higher tensile strength. Additive Group B, through depolarization and low current density control, allows 1L-B to develop coarse grains, maintaining higher elongation. Based on the combination of the two compositions and processes for 1L-A and 1L-B, polarization electrodeposition for fine-grain layers and depolarization electrodeposition for coarse-grain layers were realized. This ultimately constructed the multilayer-grain-structured 3L-ABA copper foil, achieving synergistic improvement in mechanical properties through fine-grain strengthening and coarse-grain plasticity.

## 4. Conclusions

The study achieves precise control through the differential polarization characteristics of the composite additive: Additive A promotes transient nucleation and grain refinement via strong polarization, while Additive B facilitates grain growth due to its weaker polarization effect. Based on this mechanism, by precisely designing electrochemical polarization behavior and nucleation patterns, researchers successfully fabricated 3L-ABA copper foil with layered gradient structures, achieving controllable preparation of gradient structures at ultra-thin scales. The performance characterization results demonstrate that the 3L-ABA copper foil exhibits outstanding comprehensive properties: its tensile strength reaches 604 ± 18 MPa, significantly surpassing the 289 ± 10 MPa of the control sample 1L-B copper foil; the elongation rate reaches 3.6%, markedly better than the 1.7 ± 0.2% of traditional high-strength fine-grain copper foil, while maintaining low roughness (Rz = 0.46 μm). The superior properties of 3L-ABA copper foil stem from a synergistic multi-mechanism approach: high T_C_ (111) orientation enhances material strength, while the transition orientation promotes stress homogenization for precise texture control. The surface fine grains dominate the strengthening effect, and the intermediate coarse grains induce slip to ensure plasticity. Additionally, the high geometrically necessary dislocation density (*ρ*_GND_) and twin density provide extra reinforcement, collectively achieving strength–plasticity synergy.

This research provides novel insights for designing and fabricating high-performance ultra-thin metallic materials, with broad application prospects in advanced manufacturing. Furthermore, while this study focused on the A-B-A gradient sequence to establish the principle future research could further investigate the effects of different layered sequence structures (e.g., the B-A-B-type “coarse–fine–coarse” gradient structure) on the mechanical behavior of materials, in order to achieve a more comprehensive understanding of interlayer synergistic effects and the laws governing property regulation.

## Figures and Tables

**Table 1:** Compositions of electroplating bath A and bath B.

Component	Manufacturer	Grade	Electroplating Bath A	Electroplating Bath B	City
Cu^2+^ (g/L)	Xilong Scientific	AR	70	80	Shenzhen, China
H_2_SO_4_ (mL/L)	Xilong Scientific	AR	66	66	Shenzhen, China
HCl (μL/L)	Xilong Scientific	AR	47	47	Shenzhen, China
Collagen (mg/L)	Energy Chemical	Protein > 90%	20	20	Shanghai, China
SPS (mg/L)	TCI	>97.0%	5	3	Shanghai, China
Thiourea (mg/L)	General-Reagent	AR	5	0	Shanghai, China
2M5S (mg/L)	Maclin	≥98% (HPLC)	5	0	Shanghai, China

## Data Availability

The original contributions presented in this study are included in the article. Further inquiries can be directed to the corresponding authors.
